# A Lightweight and Efficient Method of Structural Damage Detection Using Stochastic Configuration Network

**DOI:** 10.3390/s23229146

**Published:** 2023-11-13

**Authors:** Yuanming Lu, Di Wang, Die Liu, Xianyi Yang

**Affiliations:** 1 School of Information Science and Engineering, Chongqing Jiaotong University, Chongqing 400074, China; diaodiaolu@gmail.com; 2 School of Civil Engineering, Chongqing Jiaotong University, Chongqing 400074, China; liudie111@163.com (D.L.); xianyi@yahoo.com (X.Y.)

**Keywords:** sensors, multi-sensors, stochastic configuration networks, structural health monitoring, convolutional neural network, neural network pruning

## Abstract

With the advancement of neural networks, more and more neural networks are being applied to structural health monitoring systems (SHMSs). When an SHMS requires the integration of numerous neural networks, high-performance and low-latency networks are favored. This paper focuses on damage detection based on vibration signals. In contrast to traditional neural network approaches, this study utilizes a stochastic configuration network (SCN). An SCN is an incrementally learning network that randomly configures appropriate neurons based on data and errors. It is an emerging neural network that does not require predefined network structures and is not based on gradient descent. While SCNs dynamically define the network structure, they essentially function as fully connected neural networks that fail to capture the temporal properties of monitoring data effectively. Moreover, they suffer from inference time and computational cost issues. To enable faster and more accurate operation within the monitoring system, this paper introduces a stochastic convolutional feature extraction approach that does not rely on backpropagation. Additionally, a random node deletion algorithm is proposed to automatically prune redundant neurons in SCNs, addressing the issue of network node redundancy. Experimental results demonstrate that the feature extraction method improves accuracy by 30% compared to the original SCN, and the random node deletion algorithm removes approximately 10% of neurons.

## 1. Introduction

In engineering structures, structural damage represents an intrinsic impairment, shaped by both environmental and mechanical factors, with the potential to spread internally. This type of damage accelerates the aging process and diminishes the intended design lifespan of the structure, highlighting the pivotal importance of diligent monitoring. In the field of structural health monitoring (SHM), monitoring techniques have evolved from visual inspection to more advanced methods for structural health monitoring and damage detection. Various technologies have been developed for the detection, localization, and quantification of damage in structures [1,2,3]. Among them, vibration-based damage detection techniques have been extensively researched and successfully applied. These techniques assess damage in structures by recording and analyzing their vibration responses, enabling informed decisions regarding structural health [4]. Over the course of several decades, significant advancements have been achieved in this field [5,6,7]. The principle of vibration-based damage detection is based on observing changes in the vibration response characteristics of structures when subjected to external excitation. By employing suitable sensors to record the structural vibration data and applying signal processing and pattern recognition techniques, potential damage within the structure can be detected.

Damage refers to changes in the geometric shape or material properties of a structure, which have adverse effects on its performance, safety, reliability, and service life [8]. Unlike complete failure, damage refers to the deterioration of structural functionality, resulting in a decrease in performance [9,10]. If left untreated, damage accumulates gradually and eventually leads to structural failure. The type of damage determines the mode of failure, some of which develop gradually, while others occur suddenly [11]. For instance, damage caused by corrosion or fatigue typically develops slowly, whereas destruction resulting from earthquakes or fires may lead to rapid failure [12]. This difference is attributed to variations in the mode of action and the extent of impact of different types of damage.

**Figure 1:**
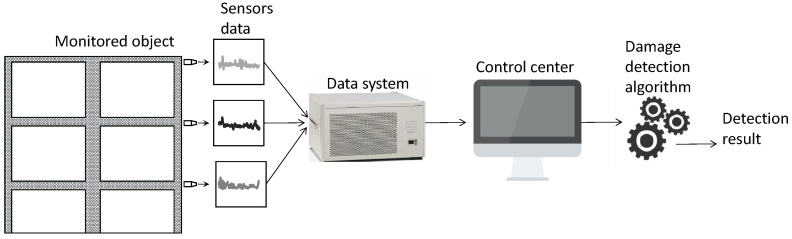
Structure diagram of damage detection systems.

In order to monitor the structural health condition, it is necessary to deploy a monitoring system on the monitored object. SHM systems have been applied in various engineering fields, such as mechanical and civil engineering [13,14,15]. The monitoring system conducts long-term measurements on the monitored object itself and its surrounding environment [16] and analyzes the data collected by sensors to locate, identify, and quantify damage [17]. A typical damage identification system is illustrated in Figure 1. A number of sensors are installed on the monitored object, with different sensors serving different purposes. For example, some are used for monitoring the vehicle load [18], while others are used for anomaly detection [19]. The monitored object can be a bridge, tunnel, building, mechanical equipment, etc. The sensors include vibration sensors, acceleration sensors, pressure sensors, temperature sensors, and so on. These sensors collect relevant data from the object under investigation, and, after undergoing operations such as data storage, transmission, and management by the data system, the data are sent to the control center. The control center performs relevant analysis using algorithms on the processed structured data [20].

With the advancement of sensor technology and machine learning techniques, data-driven monitoring methods have gained considerable attention. The development of sensors has enabled the collection of large amounts of real-time monitoring data, while machine learning techniques have provided various neural network algorithms. Leveraging machine learning, these monitoring methods can extract features from extensive real-time monitoring data and accomplish the immediate assessment and monitoring of engineering structure conditions. These methods not only automate the monitoring process, improving its accuracy and reliability, but also provide timely alerts and warnings, assisting engineers in taking appropriate maintenance and repair measures to ensure structural safety and reliability. A large number of traditional machine learning methods have been applied to structural health monitoring [21]. Traditional machine-learning-based identification methods mainly included support vector machines [22], neural networks [23,24], clustering [25,26], and principal component analysis [27]. However, these methods still rely on manual feature extraction, although they alleviate the issue of low monitoring efficiency.

In recent years, deep learning has rapidly developed, and various deep learning methods have been introduced into structural damage identification, alleviating the inefficiency of traditional machine learning methods in feature extraction [28]. Among these methods, convolutional neural networks (CNNs) and recurrent neural networks (RNNs) have been widely used [29,30,31]. Some researchers have combined CNNs and RNNs to capture the spatio-temporal features of monitoring data [32,33]. Furthermore, to enhance the attention of the model to the relationships among sensors, attention mechanisms have been introduced by some researchers [34].

Although deep-learning-based methods have improved the accuracy of damage detection, these methods overlook the operational efficiency of the entire monitoring system. In a monitoring system, numerous algorithms are integrated, and some algorithms are deployed on embedded devices with limited computational capabilities, posing significant challenges to algorithm efficiency. In addition to neglecting monitoring efficiency, the definition of neural network structures is subjective, experience-dependent, and static. Typically, a fixed network structure is defined in advance and trained on fixed data. This approach presents some issues. If the network is defined too complexly, there is a risk of overfitting and wastage of computational resources. Conversely, if the network is defined too simply, there is a risk of underfitting. For different monitoring tasks, traditional neural networks require different structure definitions based on past experience. Once a network is defined, its structure is generally not subject to alteration, and a static network configuration may not be suitable for monitoring dynamic changes in data.

Data randomization learning is a fast modeling method. Pao et al. proposed a random vector functional link network (RVFL) [35], but the RVFL does not have universal approximation capabilities in certain cases [36,37]. To address this limitation, Wang et al proposed a stochastic configuration network (SCN) [38], which is an emerging incremental learning method that dynamically increases network nodes based on previous errors and data. With the introduction of the SCN, several outstanding studies related to SCNs have gradually emerged. The strategy of “block learning” was introduced from the perspective of parallel learning by Dai et al. [39], which accelerated the training process of the SCN. Zhao et al. [40] introduced the chaotic sparrow algorithm to search for suitable hyperparameters, making the training process of SCNs more stable. Liu et al. [41] utilized statistical characteristics of vibration signals as inputs for the SCN, enabling the diagnosis of mechanical equipment faults. Li et al. [42] combined convolution with an SCN to automatically grade vegetables and fruits.

In terms of the dynamic nature of the structure, a randomly configured network can alleviate the shortcomings of previous methods in dynamic environments. However, due to its inherent nature as a fully connected neural network and the insufficient updating of network nodes compared to gradient descent, it still has its drawbacks. Firstly, in relation to the monitoring task, its input format is not user-friendly as it requires data inputs to be one-dimensional vectors. In practical scenarios, multiple sensors [18,32,43] are often deployed on the monitored object, resulting in the majority of monitoring data being two-dimensional matrices. If these matrices are directly converted into one-dimensional vectors and fed into the SCN, it can lead to loss of internal information within the data, resulting in a degraded performance of the SCN. Secondly, there are redundant nodes within the network. These extra nodes increase the number of network parameters and slow down the inference speed, which is unfavorable for monitoring systems.

To address the first issue, inspired by extreme learning machines (ELMs) [44] and random convolutional kernel transform (Rocket) [45], a feature extraction method based on random mapping is proposed. Specifically, a large number of random convolutional kernels are employed to extract features, which are independent of the backpropagation algorithm, and the parameters of the convolutional kernels are not updated. The extracted features serve as mappings in a high-dimensional space and are then fed into the SCN for recognition. To tackle the second issue, this paper presents a random node deletion algorithm that can prune redundant neurons in the SCN. Through simulation analysis, the effectiveness of the proposed algorithm is verified. Specifically, the feature extraction method proposed in this paper is capable of achieving a 30% performance enhancement for the SCN on two monitoring datasets, thereby addressing the inherent deficiency of the SCN in handling temporal data. The node pruning algorithm introduced in this paper can remove 10% of neurons within an acceptable margin of error.

## 2. Methods

In the context of structural health monitoring systems, a multitude of monitoring models are deployed, necessitating consideration not only of the accuracy of these models in monitoring tasks but also of their inferential speed. Some neural-network-based models, such as recurrent neural networks [46], while capable of accurately predicting time series monitoring data, exhibit a large number of parameters and require the manual predefinition of network architecture based on prior experience. Defining a conventional neural network that is too large results in a wasteful allocation of computational resources, whereas defining one that is too small fails to meet the performance requirements. In contrast, SCNs represent an incremental neural network approach wherein the network structure does not require manual definition but is generated on-the-fly during the training process.

### 2.1. Review of SCN

**Figure 2:**
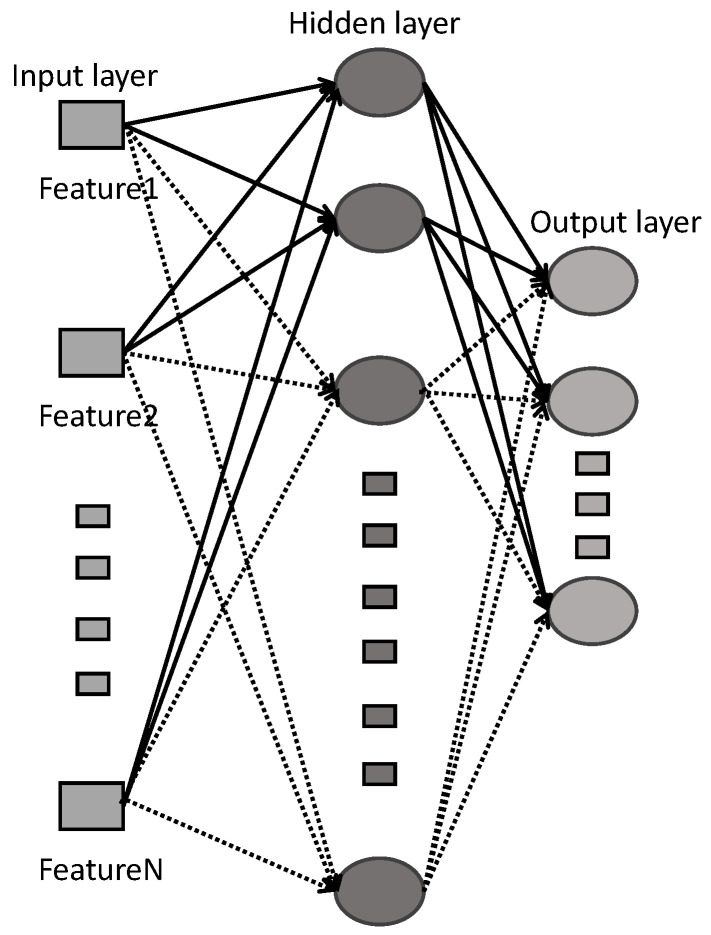
Structure diagram of stochastic configuration network.

The SCN is a neural network that has emerged in recent years that differs from traditional gradient-based neural networks. It does not rely on gradient descent algorithms and instead incrementally constructs the network structure by randomly configuring suitable network nodes. The specific structure is depicted in the accompanying Figure 2. The nodes in the intermediate hidden layer are incrementally constructed, starting with the generation of the first node, followed by the second node, and so on until the *N*-th node is generated. The solid lines represent already configured neurons, while the dashed lines represent yet-to-be-configured and generated neurons. In the following review, the main aspects of SCNs will be revisited and summarized. First, the general approximation properties of an SCN will be introduced. Given a target function $f:R^{d}\to R^{m}$, suppose that a network has been configured with L−1 hidden nodes, denoted as $f_{L-1}=\sum_{l=1}^{L-1}\beta_{l}\Phi_{l}\left(w_{l}^{T}x+b_{l}\right)\left(L=1,2,\ldots ;f_{0}=0\right)$, where $\beta_{l}={\left[\beta_{l,1},\beta_{l,2},\ldots ,\beta_{l,m}\right]}^{T}$ and $\Phi_{l}\left(w_{l}^{T}x+b_{l}\right)$ is the activation function of the *l*-th node. The residual error is defined as $e_{L-1}^{*}=f-f_{L-1}=\left[e_{L-1,1}^{*},\ldots ,e_{L-1,m}^{*}\right]$.

Let $\Gamma =\{\Phi_{1},\Phi_{2},\Phi_{3},\ldots \}$ be the set of real-valued functions and span(Γ) be the space spanned by Γ. $L_{2}\left(D\right)$ represents all Lebesgue measurable functions $f=\left[f_{1},f_{2},\ldots ,f_{m}\right]:R^{d}\to R^{m}$ defined on $D\subset R^{d}$. The $L_{2}$ norm is defined as

(1) $$∥f∥={\left(\sum_{q=1}^{m}\int_{D}{\left|f_{q}\left(x\right)\right|}^{2}dx\right)}^{2}.$$

The inner product of *f* and $\theta =[\theta_{1},\theta_{2},\ldots ,\theta_{m}]$ is defined as

(2) $$\langle f,\theta \rangle =\sum_{q=1}^{m}\langle f_{q},\theta_{q}\rangle =\sum_{q=1}^{m}\int_{D}f_{q}\left(x\right)\theta_{q}\left(x\right)dx.$$

Suppose that span(Γ) is dense in the $L_{2}$ space and holds for $\Phi \in \Gamma ,0<∥\Phi ∥<b_{\Phi }$. Given the inequality 0<r<1, as well as a non-negative sequence $\left\{\mu_{L}\right\}$ with limit $lim_{L\to \mu_{L}}=0$ and $\mu_{L}\leq \left(1-r\right)$, for L=1,2,3,…, define $\delta_{L}=\sum_{q=1}^{m}\delta_{L,q},\delta_{L,q}=\left(1-r-\mu_{L}\right)∥e_{L-1,q}∥$. If the random basis functions satisfy the inequality

(3) $${\langle e_{L-1,q},\Phi_{L}\rangle }^{2}\geq b_{\Phi }^{2}\delta_{L,q},q=1,2,\ldots ,m,$$

and the output weights of the hidden layer are

(4) $$\beta =\left[\beta_{1},\beta_{2},\ldots ,\beta_{L}\right]={\mathrm{argmin}}_{\beta }∥f-\sum_{j=1}^{L}\beta_{j}\Phi_{j}∥,$$

then $\lim_{L\to \infty }∥f-f_{L}∥=0$. After the introduction of the universal approximation property, the subsequent section presents the generation process of an SCN. Consider a training set consisting of N sample pairs $\left\{\left(x_{n},y_{n}\right),n=1,2,\ldots ,N\right\}$, where $x_{n}\in R^{d},y_{n}\in R^{m}$. Let $X\in R^{N\times d}$ and $Y\in R^{N\times m}$ represent the inputs and outputs, respectively, corresponding to these *N* samples in the network. Furthermore, let $e_{L-1}\left(X\right)\in R^{N\times M}$ denote the residual error matrix, where each column $e_{L-1,q}\left(X\right)={\left[e_{L-1,q}\left(x_{1}\right),\ldots ,e_{L-1,q}\left(x_{N}\right)\right]}^{T}\in R^{N},q=1,2,\ldots ,m$. The output vector of *X* at the *L*-th node, denoted on $\Phi_{L}$, is defined as

(5) $$h_{L}\left(X\right)=\left[\Phi \left(w_{L}^{T}x_{1}+b_{L}\right),\ldots ,\Phi \left(w_{L}^{T}x_{N}+b_{L}\right)\right]$$

Then, the output matrix of the hidden layer in $f_{L}$ can be represented as $H_{L}=\left[h_{1},h_{2},\ldots ,h_{L}\right]$. Let

(6) $$\xi_{L,q}=\frac{{\left(e_{L-1,q}^{T}\left(X\right)\cdot h_{L}\left(X\right)\right)}^{2}}{h_{L}^{T}\left(X\right)\cdot h_{L}\left(X\right)}-\left(1-r-\mu_{L}\right)e_{L-1,q}^{T}\left(X\right)e_{L-1,q}\left(X\right),q=1,2,\ldots ,m$$

be defined as an intermediate variable. Perform $T_{max}$ configurations on node $\Phi_{L}$, selecting the candidate node parameters that maximize $\xi_{L}=\sum_{q=1}^{m}\xi_{L,q}\geq 0$ as the parameters for the *L*-th node. Substitute the label matrix *Y* for the objective function in Equation (4), and then calculate Equation (4) using the least squares method.

(7) $$\beta ={\mathrm{argmin}}_{\beta }∥f-\sum_{j=1}^{L}\beta_{j}\Phi_{j}∥=H^{+}Y.$$

Here, $H^{+}$ represents the generalized inverse matrix of *H*. If the predicted results do not meet the tolerance error or the number of nodes does not reach the predetermined maximum number of nodes, the above operations are continued to generate new nodes.

As neural network node deletion is involved in this paper, the following formula is provided for calculating the number of parameters in a single-hidden-layer fully connected neural network. Assuming that the input layer has a dimension of *I*, the output layer has a dimension of *O*, and the number of neurons in the intermediate hidden layer is *L*, with each neuron having a bias term, the total number of parameters is denoted as *P*. The calculation of the parameter of network count is given by

(8) P=(I+O+1)L.

In order to investigate the computational cost of the SCN, floating-point operations per second (FLOPs) are introduced for a quantitative assessment of the SCN’s computational performance. An SCN is fundamentally a single-hidden-layer fully connected neural network with an activation function. The activation function employed in this study is the Sigmoid activation function, expressed as $S\left(z\right)=\frac{1}{1+exp(-z)}$. Through straightforward derivation, the SCN’s FLOPs can be represented as

(9) FLOPs=(2L∗(I+O)+3L)∗bs,

where bs represents the batch size, denoting the number of input samples.

### 2.2. Feature Extraction Method Based on Randomly Parameterized Rectangular Convolution

**Figure 3:**
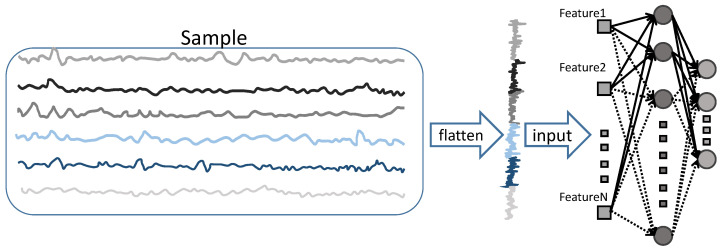
Flattening of multivariate time series: dimension mixing and information loss.

The monitoring data for structural health monitoring can be defined using multivariate time series from multiple sensors. In structural health monitoring, the focus is typically on the state and performance of structures such as bridges and buildings. To obtain information about the state of the structure, multiple sensors are commonly used to measure different physical quantities, forming a monitoring dataset. For example, *N* acceleration sensors may be used to collect signals at a frequency *f* for a duration of *T*, and these collected data are combined to form a dataset based on acceleration signals. The input of the SCN is a one-dimensional vector, while monitoring data are typically two-dimensional multivariate time series data. If the monitoring data are directly flattened into a one-dimensional format (as shown in Figure 3), the performance of the model trained on such data will be poor. This is because flattening the data disrupts or even loses the information in the temporal and channel domains. Therefore, a way needs to be found to ensure that the features of the monitoring data can be effectively extracted.

**Figure 4:**
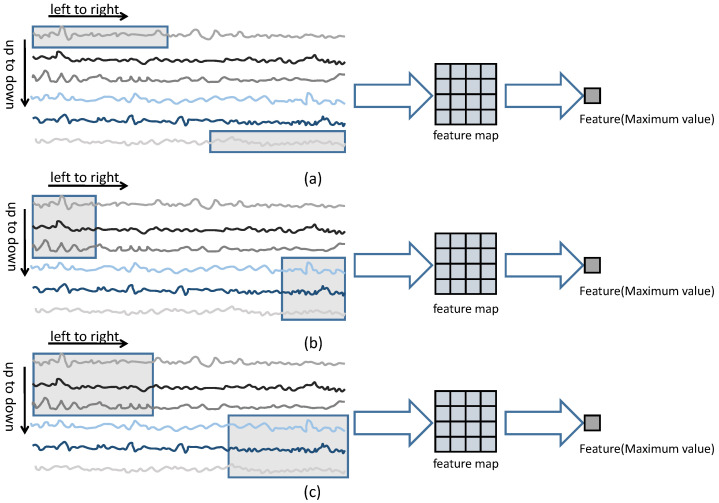
Different styles of convolution kernel for random feature extraction. (**a**) One-dimensional convolution kernel, (**b**) square convolution kernel, (**c**) rectangular convolution kernel.

**Figure 5:**
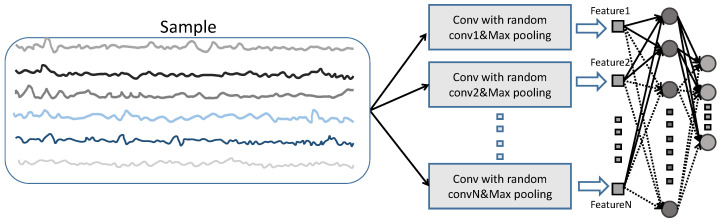
A framework for training SCN based on random convolution kernel.

An ELM [44] is a simple machine learning approach. Initially, the ELM randomly initializes the input weights of the hidden layer. Then, it linearly maps the data using these weights to a high-dimensional space. Finally, it updates the weights between the hidden layer and the output layer using the least squares method. Rocket [45] is a simple yet effective method for single-variable time series classification. Its basic idea is to use a large number of randomly generated convolutional kernels to extract features from the time series, which are then used for subsequent modeling tasks. Rocket argues that time series data are not as complex as images or natural language, and therefore do not require complex neural networks or tedious training processes. Rocket has been validated on single-variable time series data. However, the original Rocket method uses one-dimensional convolutional kernels, which may not capture the channel-domain information effectively for multivariate time series monitoring data. Inspired by ELMs and Rocket, this study introduces a feature extraction approach based on random two-dimensional convolutional kernels. Experimental results demonstrate that extending the one-dimensional kernels in Rocket to two-dimensional kernels leads to performance improvements. Traditional two-dimensional convolutions use square kernels with odd side lengths, which may be more suitable for images than monitoring data. Therefore, it is worth emphasizing that this study selects random two-dimensional convolutional kernels that are more suitable for monitoring data, with the width and height chosen from a given range of positive integers. The experimental results also confirm the effectiveness of using random rectangular kernels. The feature generation methods based on Rocket, square convolutional kernels, and rectangular convolutional kernels are illustrated in Figure 4. In all three methods, the convolutional kernels move from left to right and from top to bottom, and the features are selected by directly choosing the maximum value in the feature maps. The forward figure of the entire framework is shown in Figure 5. Given a sample, features are constructed using the approach depicted in Figure 4. N convolutional kernels are randomly generated, with each kernel generating a feature. Thus, a sample corresponds to N features. These features, generated through random convolutions, are used as inputs for the SCN. For the hyperparameters of the convolutional kernel (kernel size, input–output channel numbers, stride, padding, dilation factor, number of convolution groups, bias), a range is given. In the experiments, the convolutional kernels are randomly generated based on these specified ranges. The weight parameters of the convolutional kernels are initialized randomly using the *Kaiming uniform* [47] method. The specific configurations of the random convolutional kernels used in the experiments are provided below.

Specifically, the side length of the three convolution kernels is randomly determined by selecting integers from 1 to 10, with each integer having an equal probability of selection. The number of channels in the output feature map is set to 1. The stride, zero-padding, and dilation factors are equally and randomly determined from integers within the range of 1 to 3. The bias of the convolution kernels is randomly drawn from a uniform distribution between (−1,1). The weights are initialized using PyTorch, a deep learning framework’s built-in weight initialization method. The input features of the SCN are represented by the maximum values of the feature maps. The specific setting methods for parameters of the convolution kernel are shown in Table 1.

### 2.3. Random Node Deletion Algorithm

Given a pre-trained SCN with *L* neurons, the node deletion problem can be considered as an approximate dynamic programming problem. The original problem is defined as follows: the accuracy of the network is maximized by deleting a certain number of neurons. The state of this problem, referred to as states[n], represents the maximum accuracy achieved by deleting n nodes from the total of *L* nodes. In other words, it is necessary to compute the combination of nodes, calculate the accuracy for each combination, and select *n* nodes from the *L* nodes for deletion, resulting in $C_{L}^{n}$ possible combinations. After the nodes are deleted, the accuracy of the network is calculated and the accuracies are stored in a List. The state transition equation can be defined as follows:

(10) states[n]=max(states[n−1],max(List))

The current state *n* represents the maximum accuracy achieved among all models with *n* nodes removed, compared to the maximum accuracy achieved in the previous state. However, due to the involvement of combinatorial calculations, the time complexity is considerably high. To mitigate this, a randomized node deletion approach is employed as an approximation, aiming to reduce the time cost. It is worth emphasizing that the n removed nodes are deleted randomly, and the probability of removal is equal for each node to be deleted.

An acceptable accuracy threshold, denoted as ϵ, is set as the pruning criterion. In this study, B is defined as ϵ=α−0.01, where α represents the accuracy of the original network. This indicates that the accuracy of all pruned networks generated during the pruning process is expected to be no more than 1% lower than the accuracy of the original network.

The following pseudo-code (Algorithm 1) presents the algorithm for node deletion:
**Algorithm 1.** Node Random Pruning Algorithm.Given the parameters to be deleted, $W=[w_{1},\ldots ,w_{L}]$,$b=[b_{1},\ldots ,b_{L}]$,$\beta =[\beta_{1},\ldots ,\beta_{L}]$,where *L* is the number of node; the initial accuracy of the network, α; test data X,Y;setting an acceptable accuracy ϵ=α−0.01; and the maximum number of nodes thatcan be deleted, $N_{max}=\left\lfloor 0.2*L\right\rfloor$.**1.** Initialize $states\leftarrow zeros(N_{max}+1)$,states[0]←α,diclist←[]**2. For **n=1** To **$N_{max}+1$** Do ****3.**  $num=C_{L}^{n}$,accuracy_and_indices←{},$accuracy_{l}ist\leftarrow$[]**4.**  
**If** 
num>10000
**5.**     
num=10000
**6.**  
**For** 
k=1
 **To** 
num
 **Do**
**7.**     Randomly choice indices of *n* nodes from original nodes.**8.**     Remove the corresponding nodes from the original list      based on the indices of the nodes to be deleted.**9.**     $W^{*}=$ delete (W,indices)**10.**     $b^{*}=$ delete (b,indices)**11.**     $\beta^{*}=$ delete (β,indices)**12.**     Calculate the accuracy of the model after removing the nodes.**13.**     accuracy= accuracy_score $\left(W^{*},b^{*},\beta^{*},X,Y\right)$**14.**     **If** 
accuracy≥ϵ
**15.**      accuracy_and_indices← append {accuracy:indices}**16.**      accuracy_list← append [accuracy]**17.  End For** (corresponds to **Step 4**)**18.**  diclist← append [accuracy_and_indices]**19.**  states[n]= max (state[n−1], max (accuracy_list))**20. End For** (corresponds to **Step 2**)**21. Return** states,diclist.

In the pseudo-code, the parameter “num” represents a hyperparameter that can be appropriately adjusted as the problem scale increases. In addition to returning the approximate state matrix, the pseudo-code also returns a list that stores the model accuracy and node indices. This list can be utilized to select an appropriate model based on the stored data. For instance, in a monitoring environment with a high tolerance for errors, models with a minimal number of parameters can be preferred. Conversely, in a monitoring environment with a low tolerance for errors, models with relatively higher accuracy are prioritized.

## 3. Explanation of Experimental Dataset

To validate the effectiveness of the proposed method in this paper, experiments will be conducted using a scaled-down mode of the Heichonggou Extra Large Bridge located in Yunnan, China (hereinafter referred to as the scaled-down mode) [32], as well as benchmark public data [43]. The following section provides an overview of the basic information and loading conditions of the data.

**Figure 6:**
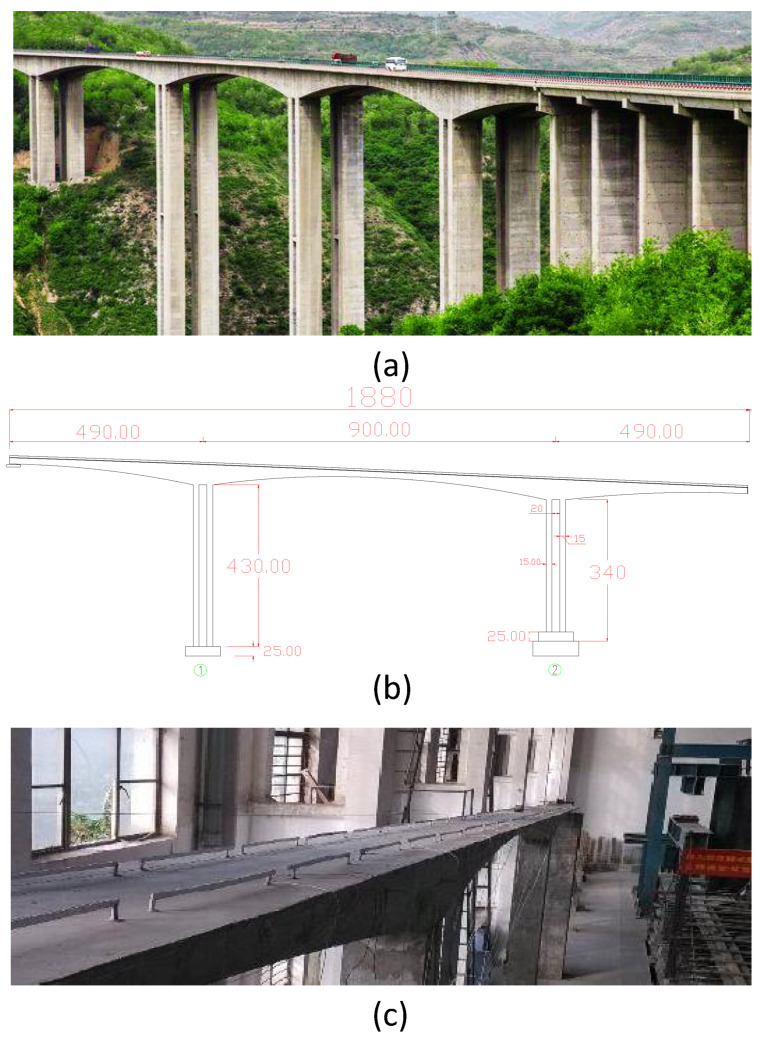
Schematic diagram of the scaled-down model. (**a**) Real scene image of the bridge; (**b**) Actual dimensions of the bridge; (**c**) Concrete model of the bridge scaled down to 1:20 ratio.

The scaled-down mode is divided into two halves, with the scaled-down mode (left half) serving as the prototype for constructing a scaled-down mode. Figure 6a shows the original image of the bridge, Figure 6b depicts a scaled image at a ratio of 20:1, and Figure 6c represents the scaled-down mode built in the laboratory. The basic structural parameters of the bridge are as follows: the superstructure of the main bridge consists of three-span prestressed concrete continuous rigid frame bridges with lengths of (98 + 180 + 98 m), and the total length of the bridge is 397 m. The box girder adopts a single-box, single-cell structure with vertical web plates. The top width of the box girder is 12.5 m, and the width of the box is 6.5 m. To simulate the structural damage and deterioration of the prototype of the Heichonggou Grand Bridge, excitation was applied at the mid-span position of the scaled-down mode based on the stress characteristics of the bridge structure. During three different static loadings applied to the mid-span of the scaled-down mode, varying degrees of cracks were observed in the structure. In the first excitation, a single transverse crack with a width of 0.06 mm and a length of 14.3 cm appeared in the mid-span bottom plate of the scaled-down mode after applying an external force of 2.889 KG. In the second excitation, two transverse cracks formed in the mid-span bottom plate of the scaled-down mode after applying an external force of 6.097 KG. The crack widths were measured to be in the range of (0.11–0.13) mm and (0.02–0.04) mm, while the crack length remained the same. In the third excitation, two transverse cracks in the mid-span bottom plate of the scaled-down mode further developed. The crack widths increased to 0.12 mm and (0.06–0.08) mm, respectively, while the crack length remained unchanged. The specific conditions for each working condition are shown in Table 2.

The second dataset used in this study is the IASC-ASCE benchmark finite element model dataset. The structural damage scenarios of the model are denoted as D1, D2, D3, D4, D5, D6, and D7, representing structural damage scenarios one to seven, respectively. The corresponding details of each structural damage scenario are presented in the Table 3 below.

The IASC-ASCE benchmark finite element model consists of six different structural damage scenarios and one initial structural condition. Each scenario is associated with 16 collected accelerometer data, with 8 sensors along the x-axis and 8 sensors along the y-axis, distributed across a four-story frame structure. Each accelerometer is used to capture the vibration signals of the model. Different structural damage states are simulated by removing supporting units or loosening bolt connections within the structure.

**Figure 7:**
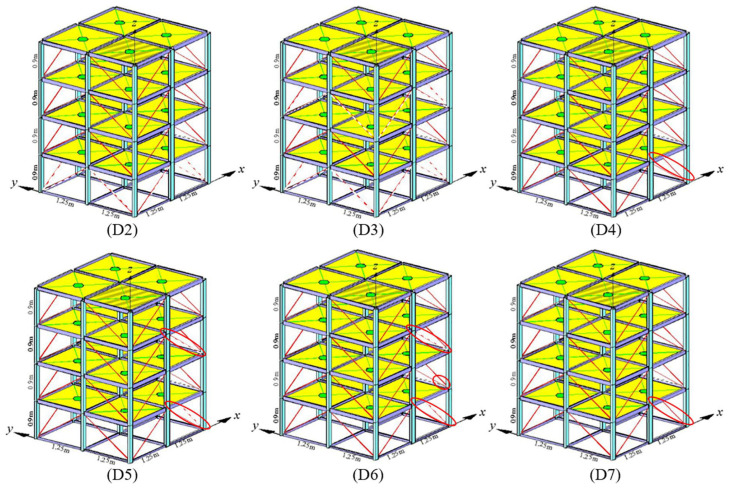
The benchmark framework.

As shown in Figure 7, the damage conditions for scenarios D2 to D7 are depicted, with each damage scenario corresponding to the conditions listed in the table. The damaged regions are highlighted with red circles and red dashed lines. Due to the simultaneous sampling of vibration signals by the 16 sensors for each scenario, the acceleration vibration data for each scenario form a multivariate time series with 16 dimensions.

In general, there are multiple sensors on the monitored object, which collect monitoring information of different dimensions; that is to say, the monitoring data usually consist of multiple dimensions, and it is necessary to analyze the correlation between different dimensions of data. From the perspective of strong correlation between sensors, a one-dimensional convolutional kernel cannot simultaneously scan the data of multiple sensors, which may result in the extracted features ignoring the similarity between them. From the perspective of weak correlation between sensors, this may also lead to the convolutional kernel neglecting the differential information between different sensors. The correlation matrices of sensors for the scaling model and the benchmark model are provided below. From the perspective of the quantitative analysis of correlation coefficients, it is necessary to choose a two-dimensional convolutional kernel.

**Figure 8:**
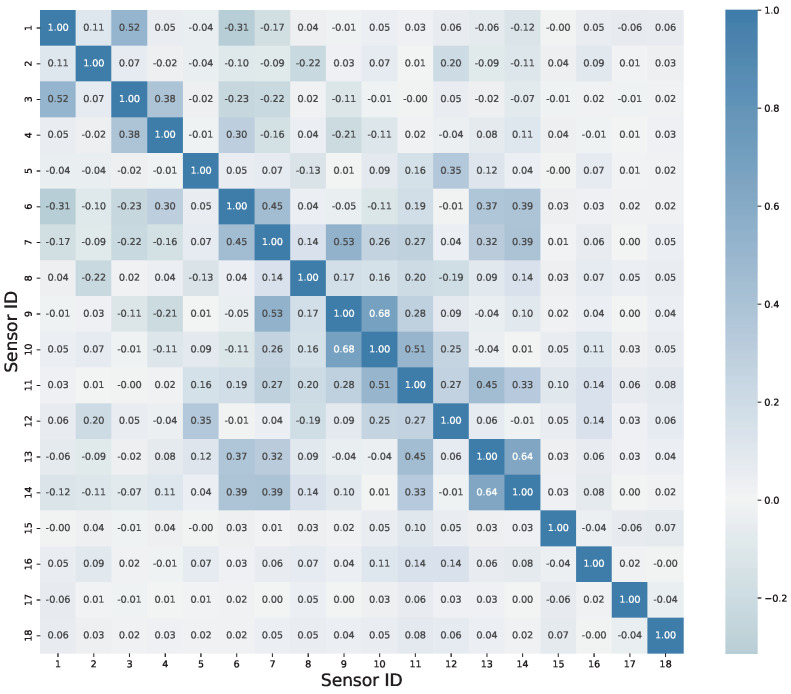
Spearman correlation matrix of sensors in scaled-down mode.

The correlation matrix of sensors for the scaled-down mode is shown in Figure 8. According to the rule of correlation strength assessment, if the correlation coefficient falls between 0.3 and 0.5, it indicates a moderate level of correlation. It can be observed that there are moderate correlations between sensors 3 and 4, 1 and 6, 6 and 7, and so on. If the correlation coefficient ranges from 0.5 to 1, it signifies a strong correlation between variables. Strong correlations can be observed between sensors 1 and 3, 7 and 9, 9 and 11, and so forth.

**Figure 9:**
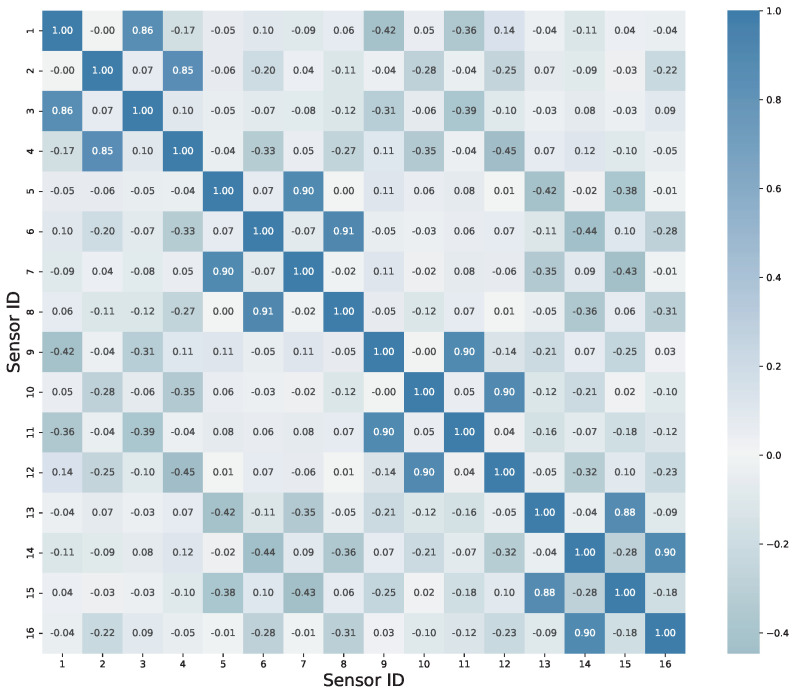
Spearman correlation matrix of sensors in benchmark model.

**Figure 10:**
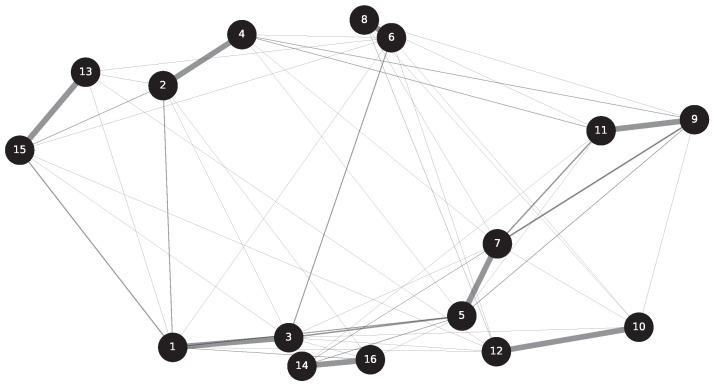
Network graph of node correlations.

The correlation matrix of sensors for the benchmark model is shown in Figure 9, providing quantitative measures of correlation. Due to the smaller size of the benchmark model compared to the scaling model, the sensors are more densely distributed, and they exhibit a higher degree of correlation. Whether it is a monitoring object with densely distributed sensors or loosely distributed sensors, there are always sensors within these objects that exhibit strong correlation. Therefore, when extracting features, it is important to consider extracting features from multiple sensors simultaneously. Although the correlation matrix graph provides specific values of correlation coefficients, it may not be user-friendly for engineers who are interested in identifying sensors with strong correlations. To address this, a novel network graph approach for determining correlations is presented below. Figure 10 illustrates a network graph depicting the correlations of benchmark among sensors of benchmark model. The black nodes with assigned numbers represent the corresponding sensor IDs. Researchers can rapidly and qualitatively assess the correlation between sensors based on the thickness and length of the connecting edges. The shorter and thicker the lines in the figure, the stronger the correlation among the sensors indicated. Referring to Figure 9, taking the strongest correlation between sensor 8 and sensor 6 as an example, the two most strongly associated points can be quickly identified in the graph. Without the assistance of Figure 10, this identification process would be considerably slower. Therefore, the introduction of such a network graph is deemed necessary.

## 4. Experiments

In the benchmark, an arrangement of 16 accelerometer sensors was set up within the structural framework. Thus, the dimensionality of the monitoring data collected by these 16 sensors is 16. A random selection of data, using a window of length 60, was performed for each working condition, collecting 500 samples for each. In total, 3500 samples were collected. Experimental validation indicated that both longer sample lengths and a greater number of samples (beyond 500) had negligible effects on the model’s performance, as excessively long samples could slow down the training of the model. The 3500 collected samples were divided into training and testing sets in a 7.5:2.5 ratio. For the scaled-down model with 18 sensors, samples of length 60 were also taken, but there were 1000 samples for each category, resulting in a total of 4000 samples. These were similarly divided into training and testing sets at a 7.5:2.5 ratio.

### 4.1. Experiments on the Random Feature Extraction Algorithm

**Figure 11:**
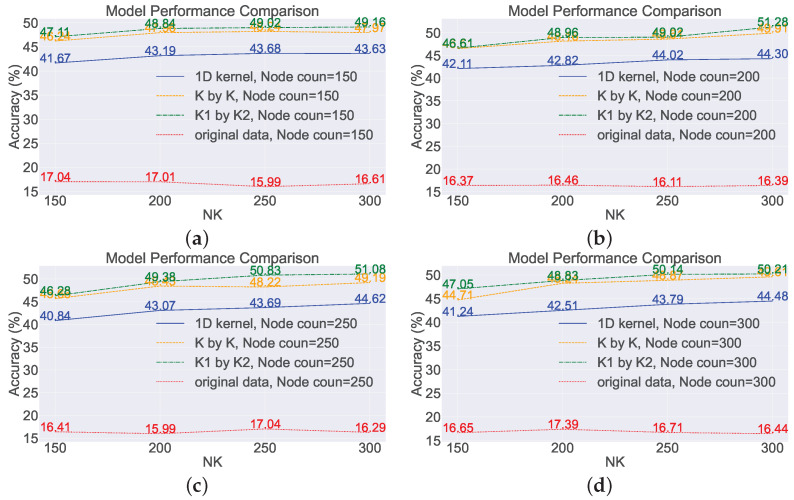
Analyzing the impact of the count of nodes on the performance of the model on the benchmark dataset. (**a**) Node count: 150; (**b**) node count: 200; (**c**) node count: 250; (**d**) node count: 300.

**Figure 12:**
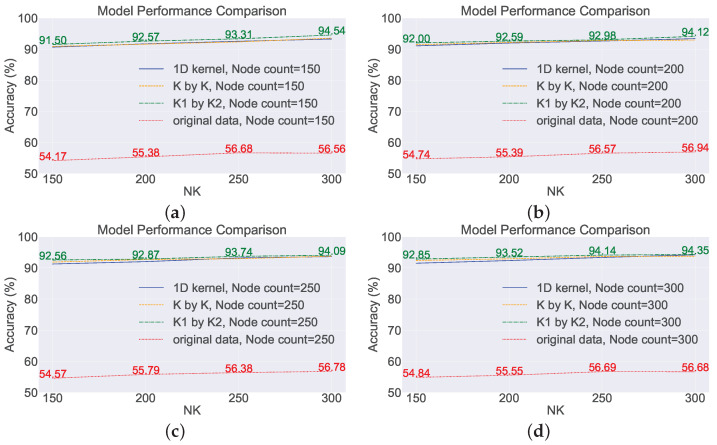
Analyzing the impact of the count of nodes on the performance of the model on the scaled-down model dataset. (**a**) Node count: 150; (**b**) node count: 200; (**c**) node count: 250; (**d**) node count: 300.

To examine the impact of the number of network nodes on model performance, four scenarios were selected with node numbers of 150, 200, 250, and 300, while controlling the number of features, NK, to be 150, 200, 250, and 300, respectively. In other words, in this section of the experiment, there are three variables: maximum node number, number of convolutional kernels, and types of convolutional kernels. By manipulating these variables, the accuracy of the model was observed (based on the average of 20 repetitions). The experiments were conducted on two datasets, and the results are shown in Figure 11 and Figure 12. The subplots in the figures represent the accuracy of different models trained with varying numbers of convolutional kernels.

Taking subplot (a) as an example, the number of convolutional kernels, NK, is set to 150, which means that the input dimension of the network is 150. The red line represents the data without any transformations directly fed into the model, while the lines of other colors represent the data after feature extraction before being fed into the model. It can be observed that directly concatenating the raw sensor data and feeding them into the network for training yields poor results, with an accuracy of only about ten percent. However, after feature extraction using random convolutional kernels, the overall model performance improved by approximately 30%. In general, as long as random convolutional kernels are used, the model performance will be improved compared to the original SCN.

Specifically, among the three feature extraction methods, the model using rectangular convolutional kernels exhibited the best performance, followed by the model using square convolutional kernels with a slight decrease in performance compared to the rectangular kernel model. The model using one-dimensional convolutional kernels showed a larger decrease in performance compared to the rectangular kernel model.

Examining the red lines in the four subplots, these lines fluctuate around 16% without any clear trend. For a seven-classification model, achieving a performance of only 16% after training indicates that the model performs poorly in the prediction task. The main reasons for the low model performance are insufficient model complexity, poor data quality, inadequate feature extraction, and overfitting. The number of nodes is varied, i.e., the complexity of the network changes, but the overall model performance does not show a changing trend, which suggests that the low predictive performance is not due to insufficient model complexity.

Based on the performance of the model on the training set during training, overfitting is not evident, as the accuracy of the model on the training set remains consistently low. The reliability of the benchmark task data can be ensured, but directly inputting the raw data into the SCN leads to a deterioration in data quality. From the lines of different colors, it can be seen that after introducing feature extraction methods, the performance of the SCN improves significantly, and the accuracy of the model can increase with increasing complexity. From this perspective, it can also be concluded that the main reason for the poor performance of the SCN on monitoring data is its inability to effectively extract features from the monitoring data.

Therefore, for the original SCN, changing the number of nodes has little impact on performance. This is because the operation of directly concatenating the raw data together as input leads to the loss of information between different sensors. Once the distribution of data is disrupted, different classes of samples will lack separability, and the compactness among samples of the same class will decrease. However, for models that incorporate feature extraction methods, the performance shows an improvement of around 30% in terms of accuracy, and, in terms of trend, the performance of model can increase with an increase in the number of nodes.

**Figure 13:**
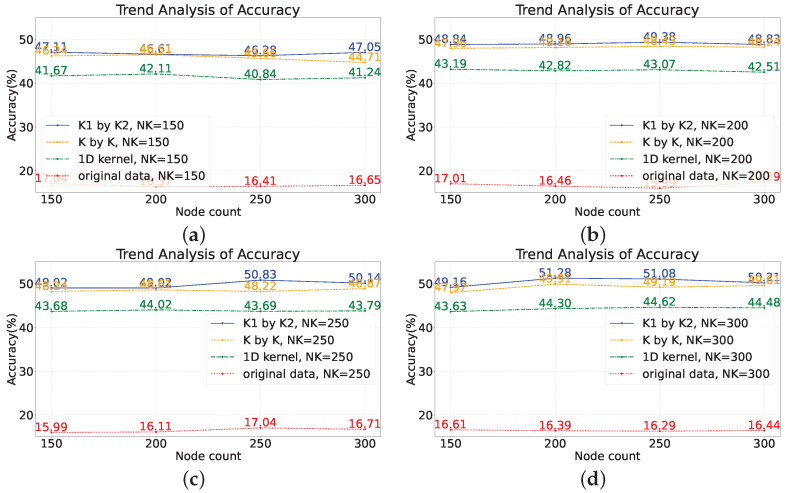
Influence of K number (NK) on model performance in benchmark dataset. (**a**) NK: 150; (**b**) NK: 200; (**c**) NK: 250; (**d**) NK: 300.

**Figure 14:**
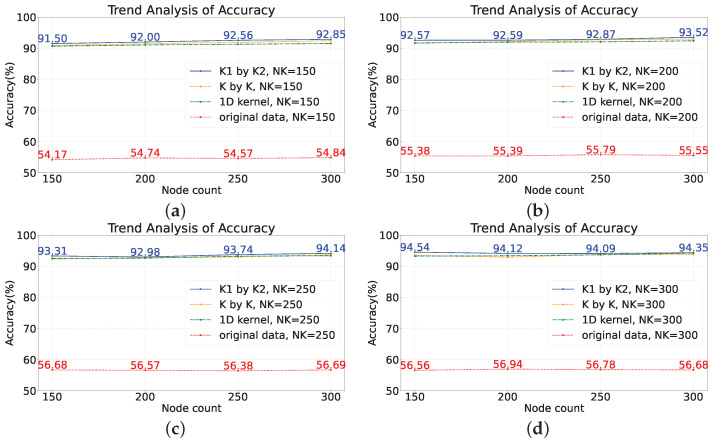
Influence of K number (NK) on model performance in scaled-down bridge dataset. (**a**) Node count: 150; (**b**) node count: 200; (**c**) node count: 250; (**d**) node count: 300.

In order to investigate the influence of the hyperparameter *K* on the model, four scenarios were selected with the number of features, *K*, set to 150, 200, 250, and 300. Similar to previous experiments, four sets of experiments were conducted with the number of nodes set to 150, 200, 250, and 300, respectively. The validation was performed on both the benchmark dataset and the scaled-down model, as shown in Figure 13 and Figure 14. From the two plots, it can be observed that, under the same value of *K*, the performance of the proposed random rectangular convolutional kernel is the best. On the test data of the scaled-down model, the detection accuracy increases as *K* increases.

However, the feature-based SCN is more sensitive to the hyperparameter *K*. Although the accuracy does not increase with the increase in *K*, it has been proven to be a hyperparameter that can alter the performance of the model.

Furthermore, on the scaled-down dataset, the performance of the original SCN exhibits a clear trend with the variation in *K*. Overall, after introducing the feature extraction method, the accuracy of the SCN is no longer in a nearly constant state regardless of how the hyperparameters are changed. As *K* increases, the performance of the model also improves.

**Figure 15:**
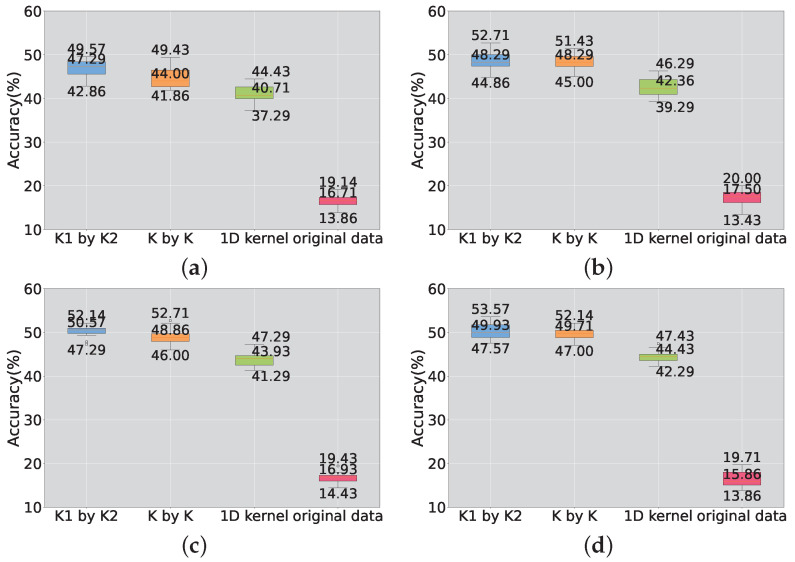
Box plot of benchmark accuracy. (**a**) The number of K is 150; (**b**) the number of K is 200; (**c**) the number of K is 250; (**d**) the number of K is 300.

The accuracy obtained from 20 training runs was utilized to construct a box plot for analysis, as shown in Figure 15, depicting the results on the benchmark dataset. This further confirms the influence of introducing feature extraction methods on the accuracy of the model. It can be observed that the box representing the accuracy of models trained on raw data is relatively low, indicating a lower level of fluctuation in accuracy compared to models trained on feature-extracted data. Regardless of the model type, the achieved accuracy is not considered high, suggesting that the benchmark task is relatively challenging. In terms of performance improvement, this approach offers a solution to enhancing the low accuracy of SCNs in complex monitoring tasks.

**Figure 16:**
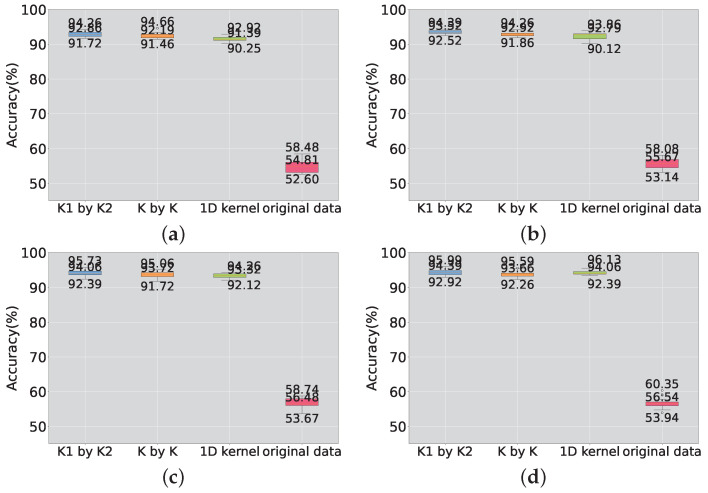
Box plot of scaled-down model accuracy. (**a**) The number of K is 150; (**b**) the number of K is 200; (**c**) the number of K is 250; (**d**) the number of K is 300.

The box plot on the scaled-down model is shown in Figure 16. It can be observed that the introduction of feature extraction methods on the scaled-down model data leads to a more stable training process and a smaller fluctuation range in model performance compared to the original SCN. In comparison to the benchmark task, the proposed method demonstrates stability on the monitoring data of the scaled-down model. It is evident that the accuracy of the model exceeds 90% when using the proposed method. In situations where the performance of the model is high, training stability is crucial. A large fluctuation in the model can easily lead to local optima. The defect of the tendency to converge to local optima has led to an increase in the training cost required for the model to achieve optimal performance.

In summary, this section validates the effectiveness of the proposed method on different types of datasets. The proposed method maximizes model performance in more challenging monitoring tasks. Additionally, in easier monitoring tasks, the proposed method not only improves performance but also enhances training stability.

### 4.2. Experiments on the Random Node Deletion Algorithm

In the experiments of this section, the convolutional kernel pattern was set as rectangular kernels, with 200 features and 250 neurons. Based on the aforementioned settings, experiments were conducted on two datasets.

#### 4.2.1. Experiments on the Random Node Deletion Algorithm in the Benchmark Dataset

**Figure 17:**
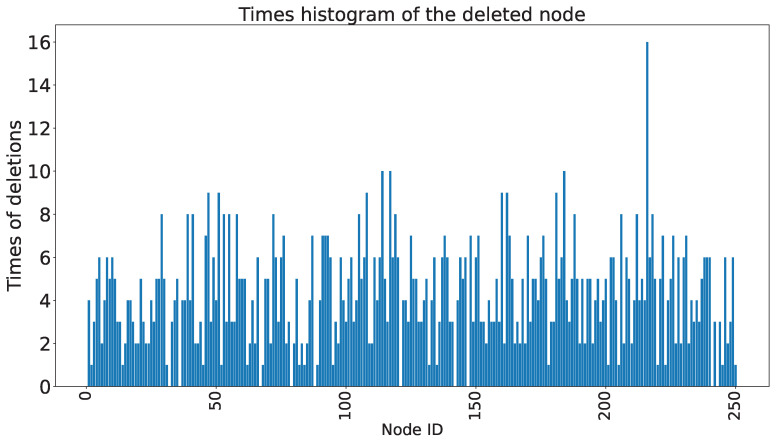
The frequency of deleted nodes (Experiments based on the Benchmark dataset).

**Figure 18:**
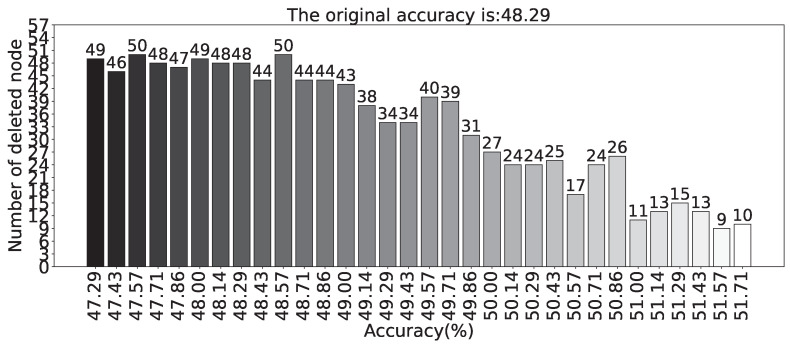
The relationship between the accuracy and the number of deleted nodes after the completion of the random node deletion algorithm (Experiments based on the Benchmark dataset).

Figure 17 presents a statistical analysis of the frequency of deleted nodes in the model when applying the random deletion algorithm. The x-axis represents the node numbers in the hidden layer, while the y-axis represents the corresponding frequency of nodes being deleted. It can be observed that some nodes have a high frequency of being deleted, indicating the presence of relatively direct redundant neurons in the network. Further experiments revealed that removing these highly deleted neurons individually does not significantly affect the performance of the network and may even lead to improvements. Figure 18 presents the statistics of the threshold-satisfied models in the random node deletion algorithm. The x-axis represents the accuracy of the model, while the y-axis represents the number of neurons deleted relative to the original model. The title indicates that the accuracy of the original model before node deletion is 48.29%. It can be observed that during the execution of the entire random node deletion algorithm, the highest accuracy reaches 51.71%, with a deletion of 10 neurons relative to the original network. The accuracy improvement of 3.42% relative to the original accuracy is achieved. Considering the input dimension of 200, neuron count of 250, and output dimension of 7, the reduction in network parameters is calculated as 2080 based on Equation (8).

In the algorithm, the upper limit for neuron deletion is set to 20% of the original network, allowing for a maximum deletion of 50 neurons. Given an acceptable accuracy, among the models that meet the ϵ, as Figure 18 shows, over half of them have deleted at least 30 neurons, and over one-third have deleted at least 40 neurons. When using an SCN for damage identification on this challenging benchmark dataset, a considerable number of redundant neurons are present. This method effectively eliminates these redundant nodes. Many models have deleted more than 45 neurons, indicating that the limit of algorithm is not restricted to 20%. Hence, when performing node deletion on an SCN exhibiting high redundancy, it is possible to set a larger value for the upper limit.

#### 4.2.2. Experiments on the Scaled-Down Model

**Figure 19:**
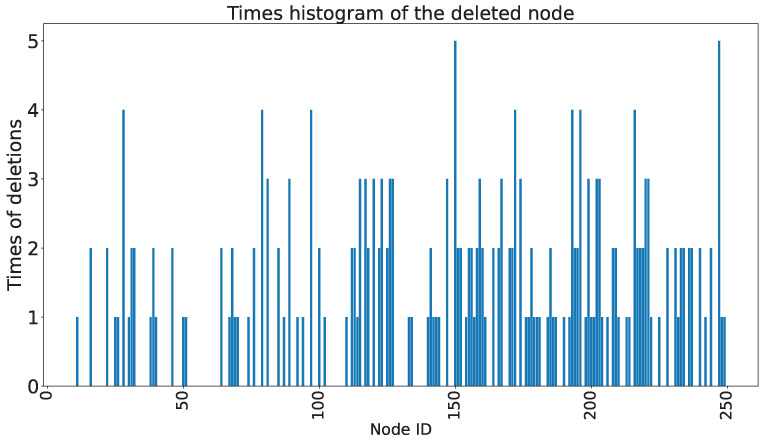
The frequency of deleted nodes (Experiments based on the scaled-down model dataset).

**Figure 20:**
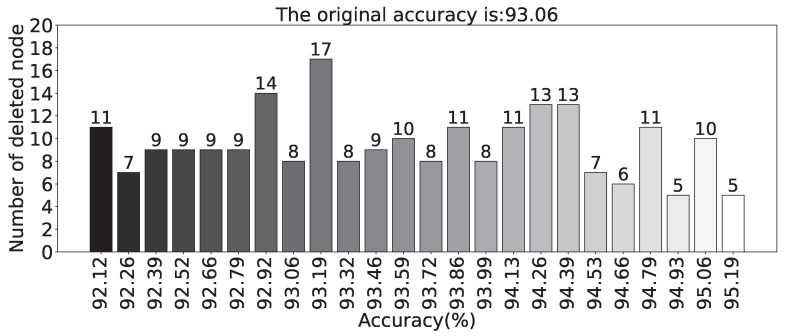
The relationship between the accuracy and the number of deleted nodes after the completion of the random node deletion algorithm (Experiments based on the scaled-down model dataset).

The frequency of deleted nodes in the model during the execution of the random deletion algorithm is presented in Figure 19. The horizontal axis represents the node index in the hidden layer, and the vertical axis represents the number of times the corresponding node was deleted. Compared to Figure 17, Figure 19 appears to be more sparse. In other words, in the experiments conducted on the scaled-down model, the degree of node redundancy is not higher than that of the models trained on the benchmark dataset. This is because the data in the scaled-down model are relatively easier to learn compared to the benchmark dataset, resulting in a higher effectiveness of nodes generated by the SCN and lower node redundancy. Despite the lower node redundancy, the random node deletion algorithm still removes some neurons while ensuring the accuracy of the model. Figure 20 shown displays the accuracy of the selected models and the corresponding number of deleted nodes after executing the random node deletion algorithm. Without any node being deleted, the original accuracy of the model is 93.06%. In the case of maximum accuracy optimization, five neurons are deleted, resulting in a reduction of 1040 parameters. With a 0.13% improvement in accuracy, 17 nodes are deleted, and the parameter count is reduced by 3536. On average, the SCN in the experiments on the scaled-down model can delete 10 neurons.

### 4.3. FLOPs Analysis of SCN

**Figure 21:**
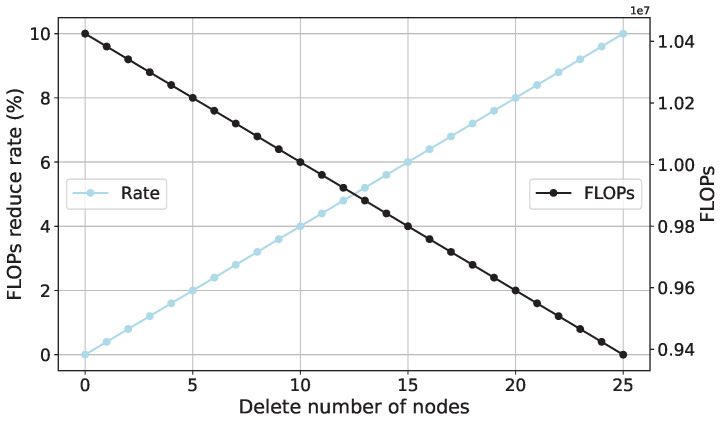
The relationship between the number of deleted nodes and SCN FLOPs (based on the benchmark dataset).

**Figure 22:**
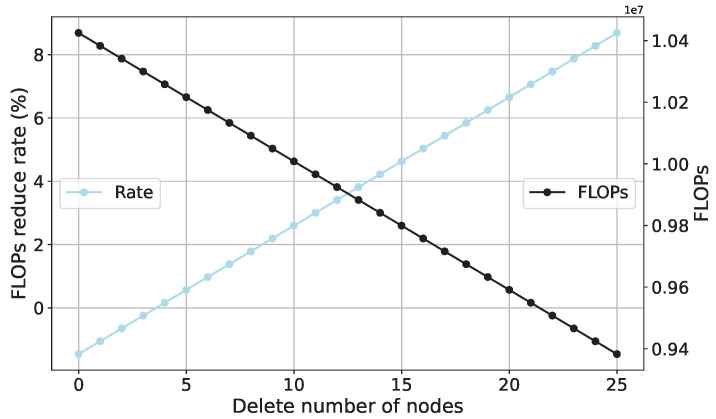
The relationship between the number of deleted nodes and SCN FLOPs (based on the scaled-down model dataset).

In accordance with the FLOPs computation Formula (9) for the SCN, the influence of node removal on FLOPs is visualized. In general, the dimensions of the input layer and output layer of the SCN are kept constant, i.e., the values of I and O remain unchanged, while the number of neurons in the hidden layer, denoted as L, is varied. Given that this study encompasses two datasets, each dataset identifies a different number of operating conditions. First, an analysis of FLOPs is conducted based on the benchmark dataset. Specifically, the input layer length of the SCN is set to 200, the number of neurons in the hidden layer is set to 250, and the output layer length is set to 7, with a batch size of 100. To observe the changes in FLOPs before and after the removal of 10% of neurons, a reduction of 10% in neurons is applied to the initial 250 neurons. As depicted in Figure 21, after removing 10% of the neurons, FLOPs decrease by approximately 10%. More precisely, after removal, the floating-point computations of the SCN are reduced by around one million. Subsequently, an analysis of FLOPs is carried out based on the downsampled model dataset, with the only alteration being the output layer length set to 4. All other settings remain consistent with the experiments based on the benchmark dataset. Analyzing Figure 22 leads to the same conclusion, where the removal of 10% of neurons results in a reduction of approximately one million floating-point computations.

## 5. Conclusions

1. Key Findings: The study’s primary findings can be summarized in three key points:
  a. Using raw multisensor data directly as input for the self-constructing network (SCN) is unsuitable.
  b. Employing randomly generated rectangular convolutional kernels for feature extraction is effective.
  c. SCNs contain redundant nodes, and the random node deletion algorithm efficiently eliminates them.
2. Feature Extraction Enhancement: In traditional deep learning, a fully connected network handles classification by extracting features. SCNs operate similarly but lack robust feature extraction capabilities for complex monitoring data. To address this, we introduce a convolution-based feature extraction approach inspired by deep learning, utilizing randomly generated rectangular convolutional kernels. This method is validated through experimental results.
3. Improved Model Performance: Concatenating raw data as input leads to information loss between sensors and low accuracy. The introduction of feature extraction using random convolutional kernels significantly improves model performance, with accuracy increasing by approximately 30%. Notably, rectangular convolutional kernels outperform one-dimensional kernels similar to Rocket.
4. Random Node Deletion: The experiments with the random node deletion algorithm demonstrate its potential for enhancing model performance, particularly when used on benchmark datasets. This approach achieves parameter reduction and improved performance.
5. Overall Implications: The experimental results emphasize the impact of employing random feature extraction, adjusting convolutional kernels, and node deletion on model performance. These findings are valuable for optimizing model design and parameter selection in monitoring data processing.
6. SCN for Multisensor Data: Conventional SCNs are not suitable for multisensor monitoring data. The study introduces random 2D convolutional kernels for feature extraction in this context. Future research should explore improved feature extraction methods and enhanced random mapping approaches.
7. Future Applications: While this paper primarily focuses on loss recognition, the framework has the potential to extend to other monitoring tasks, such as anomaly detection, remaining useful life prediction, and vehicle load modeling.
8. The method proposed in this paper is better suited for data with small mean fluctuations (such as acceleration data oscillating around zero), and is not applicable to data where mean values suddenly increase or decrease, such as deflection and strain [48]. When sampling from non-Gaussian or mixed probability distributions, it is relatively easier to collect data with skewed characteristics, heavy tails, and the presence of outliers as compared to the Gaussian distribution. This poses a significant challenge for time series models. Subsequent research will consider more universally applicable models for data of different distributions.

## Figures and Tables

**Table 1:** The range of values for convolutional kernel parameters.

Parameters	1×K	K×K	$K_{1}\times K_{2}$
Kernel size	randint [1, 10]	randint [1, 10]	randint [1, 10]
I/O channels	1	1	1
Stride	randint [1, 3]	randint [1, 3]	randint [1, 3]
Padding	randint [1, 3]	randint [1, 3]	randint [1, 3]
Dilation	randint [1, 3]	randint [1, 3]	randint [1, 3]
Groups	1	1	1
Bias	u(−1,1)	u(−1,1)	u(−1,1)
Weight	Kaiminguniform	Kaiminguniform	Kaiminguniform

**Table 2:** Description of scaled-down bridge dataset damage states.

Case	Descriptions
D1	No damage in the bridge structure.
D2	One crack with width of 0.06 mm.
D3	Two cracks in the mid-span of scale model with widths of (0.11–0.13) mm and (0.02–0.04) mm, respectively.
D4	Two cracks in the mid-span of scale model with widths of 0.12 mm and (0.06–0.08) mm, respectively.

**Table 3:** Description of IASC-ASCE benchmark finite element model dataset damage states.

Case	Descriptions
D1	No damage.
D2	Remove all diagonal supports on the first floor.
D3	Remove all diagonal supports on the first and second floors.
D4	Remove one diagonal support on the first floor.
D5	Remove a diagonal support on the first and third floors.
D6	D4 + Weaken the left side of the 18 element (first layer beam element).
D7	Reserve 2/3 of an oblique support area on the first floor.

## References

[1] Chesné S., Deraemaeker A. (2013). Damage localization using transmissibility functions: A critical review. Mech. Syst. Signal Process..

[2] Amezquita-Sanchez J.P., Adeli H. (2016). Signal processing techniques for vibration-based health monitoring of smart structures. Arch. Comput. Methods Eng..

[3] Meruane V., Heylen W. (2011). An hybrid real genetic algorithm to detect structural damage using modal properties. Mech. Syst. Signal Process..

[4] Wu R.T., Jahanshahi M.R. (2020). Data fusion approaches for structural health monitoring and system identification: Past, present, and future. Struct. Health Monit..

[5] Yang J., Xiang F., Li R., Zhang L., Yang X., Jiang S., Zhang H., Wang D., Liu X. (2022). Intelligent bridge management via big data knowledge engineering. Autom. Constr..

[6] Li R., Mo T., Yang J., Li D., Jiang S., Wang D. (2021). Bridge inspection named entity recognition via BERT and lexicon augmented machine reading comprehension neural model. Adv. Eng. Inform..

[7] Yang J., Huang L., Tong K., Tang Q., Li H., Cai H., Xin J. (2023). A Review on Damage Monitoring and Identification Methods for Arch Bridges. Buildings.

[8] Cosenza E., Manfredi G. (2000). Damage indices and damage measures. Prog. Struct. Eng. Mater..

[9] Kaouk M., Zimmerman D.C. (1994). Structural damage assessment using a generalized minimum rank perturbation theory. AIAA J..

[10] Zimmerman D.C., Kaouk M. (1994). Structural damage detection using a minimum rank update theory. J. Vib. Acoust. Trans. ASME.

[11] Farrar C.R., Worden K. (2007). An introduction to structural health monitoring. Philos. Trans. R. Soc. A Math. Phys. Eng. Sci..

[12] Farrar C.R., Worden K. (2012). Structural Health Monitoring: A Machine Learning Perspective.

[13] Avci O., Abdeljaber O., Kiranyaz S., Hussein M., Inman D.J. (2018). Wireless and real-time structural damage detection: A novel decentralized method for wireless sensor networks. J. Sound Vib..

[14] Chaabane M., Hamida A.B., Mansouri M., Nounou H.N., Avci O. Damage detection using enhanced multivariate statistical process control technique. Proceedings of the 2016 17th International Conference on Sciences and Techniques of Automatic Control and Computer Engineering (STA).

[15] Catbas F.N., Celik O., Avci O., Abdeljaber O., Gul M., Do N.T. (2017). Sensing and monitoring for stadium structures: A review of recent advances and a forward look. Front. Built Environ..

[16] Park S., Yun C.B., Roh Y., Lee J.J. (2006). PZT-based active damage detection techniques for steel bridge components. Smart Mater. Struct..

[17] Farrar C.R., Doebling S.W., Nix D.A. (2001). Vibration–based structural damage identification. Philos. Trans. R. Soc. Lond. Ser. A Math. Phys. Eng. Sci..

[18] Xia Y., Jian X., Yan B., Su D. (2019). Infrastructure safety oriented traffic load monitoring using multi-sensor and single camera for short and medium span bridges. Remote Sens..

[19] Hu L., Bao Y., Sun Z., Meng X., Tang C., Zhang D. (2023). Outlier Detection Based on Nelder-Mead Simplex Robust Kalman Filtering for Trustworthy Bridge Structural Health Monitoring. Remote Sens..

[20] Adewuyi A., Wu Z. (2006). Vibration-Based Structural Health Monitoring Technique Using Statistical Features from Strain Measurements. J. Eng. Appl. Sci..

[21] Noori Hoshyar A., Rashidi M., Yu Y., Samali B. (2023). Proposed Machine Learning Techniques for Bridge Structural Health Monitoring: A Laboratory Study. Remote Sens..

[22] Li J., Yang C., Chen J. (2023). Sound Damage Detection of Bridge Expansion Joints Using a Support Vector Data Description. Sensors.

[23] Osornio-Rios R.A., Amezquita-Sanchez J.P., Romero-Troncoso R.J., Garcia-Perez A. (2012). MUSIC-ANN analysis for locating structural damages in a truss-type structure by means of vibrations. Comput.-Aided Civ. Infrastruct. Eng..

[24] Jayaswal P., Verma S.N., Wadhwani A.K. (2010). Application of ANN, fuzzy logic and wavelet transform in machine fault diagnosis using vibration signal analysis. J. Qual. Maint. Eng..

[25] Diez A., Khoa N.L.D., Makki Alamdari M., Wang Y., Chen F., Runcie P. (2016). A clustering approach for structural health monitoring on bridges. J. Civ. Struct. Health Monit..

[26] Alamdari M.M., Rakotoarivelo T., Khoa N.L.D. (2017). A spectral-based clustering for structural health monitoring of the Sydney Harbour Bridge. Mech. Syst. Signal Process..

[27] Malhi A., Gao R.X. (2004). PCA-based feature selection scheme for machine defect classification. IEEE Trans. Instrum. Meas..

[28] Wang Di Y.S.X. (2022). Intelligent Feature Extraction, Data Fusion and Detection of Concrete Bridge Cracks: Current Development and Challenges. Intell. Robot..

[29] Tang Q., Zhou J., Xin J., Zhao S., Zhou Y. (2020). Autoregressive model-based structural damage identification and localization using convolutional neural networks. KSCE J. Civ. Eng..

[30] Xu Y., Wei S., Bao Y., Li H. (2019). Automatic seismic damage identification of reinforced concrete columns from images by a region-based deep convolutional neural network. Struct. Control Health Monit..

[31] Abdeljaber O., Avci O., Kiranyaz M.S., Boashash B., Sodano H., Inman D.J. (2018). 1-D CNNs for structural damage detection: Verification on a structural health monitoring benchmark data. Neurocomputing.

[32] Yang J., Zhang L., Chen C., Li Y., Li R., Wang G., Jiang S., Zeng Z. (2020). A hierarchical deep convolutional neural network and gated recurrent unit framework for structural damage detection. Inf. Sci..

[33] Yang J., Yang F., Zhou Y., Wang D., Li R., Wang G., Chen W. (2021). A data-driven structural damage detection framework based on parallel convolutional neural network and bidirectional gated recurrent unit. Inf. Sci..

[34] Liao S., Liu H., Yang J., Ge Y. (2022). A channel-spatial-temporal attention-based network for vibration-based damage detection. Inf. Sci..

[35] Pao Y.H., Takefuji Y. (1992). Functional-link net computing: Theory, system architecture, and functionalities. Computer.

[36] Gorban A.N., Tyukin I.Y., Prokhorov D.V., Sofeikov K.I. (2016). Approximation with random bases: Pro et contra. Inf. Sci..

[37] Li M., Wang D. (2017). Insights into randomized algorithms for neural networks: Practical issues and common pitfalls. Inf. Sci..

[38] Wang D., Li M. (2017). Stochastic configuration networks: Fundamentals and algorithms. IEEE Trans. Cybern..

[39] Dai W., Li D., Zhou P., Chai T. (2019). Stochastic configuration networks with block increments for data modeling in process industries. Inform. Sci..

[40] Zhang C., Ding S. (2021). A stochastic configuration network based on chaotic sparrow search algorithm. Knowl.-Based Syst..

[41] Liu J., Hao R., Zhang T., Wang X. (2021). Vibration fault diagnosis based on stochastic configuration neural networks. Neurocomputing.

[42] Li W., Tao H., Li H., Chen K., Wang J. (2019). Greengage grading using stochastic configuration networks and a semi-supervised feedback mechanism. Inf. Sci..

[43] Johnson E.A., Lam H.F., Katafygiotis L.S., Beck J.L. (2004). Phase I IASC-ASCE structural health monitoring benchmark problem using simulated data. J. Eng. Mech..

[44] Huang G.B., Zhu Q.Y., Siew C.K. (2006). Extreme learning machine: Theory and applications. Neurocomputing.

[45] Dempster A., Petitjean F., Webb G.I. (2020). ROCKET: Exceptionally fast and accurate time series classification using random convolutional kernels. Data Min. Knowl. Discov..

[46] Zhao H.W., Ding Y.L., Li A.Q., Chen B., Wang K.P. (2023). Digital modeling approach of distributional mapping from structural temperature field to temperature-induced strain field for bridges. J. Civ. Struct. Health Monit..

[47] He K., Zhang X., Ren S., Sun J. Delving deep into rectifiers: Surpassing human-level performance on imagenet classification. Proceedings of the IEEE International Conference on Computer Vision.

[48] Zhao H., Ding Y., Meng L., Qin Z., Yang F., Li A. (2023). Bayesian Multiple Linear Regression and New Modeling Paradigm for Structural Deflection Robust to Data Time Lag and Abnormal Signal. IEEE Sens. J..

